# An Evaluation of the Performance of OpenAI-o1 and GPT-4o in the Japanese National Examination for Physical Therapists

**DOI:** 10.7759/cureus.76989

**Published:** 2025-01-06

**Authors:** Shogo Sawamura, Kengo Kohiyama, Takahiro Takenaka, Tatsuya Sera, Tadatoshi Inoue, Takashi Nagai

**Affiliations:** 1 Department of Rehabilitation, Heisei College of Health Sciences, Gifu, JPN

**Keywords:** gpt-4o, japanese national examination for physical therapists, large language models, openai-o1, physical therapy

## Abstract

Background and objective

Recent advancements in large language models (LLMs) have expanded their applications in medical and healthcare settings. LLMs have demonstrated high performance in various national examinations for healthcare professionals. Open Artificial Intelligence Model Version 1 (OpenAI-o1) attained remarkable accuracy in the Japanese National Examination for Medical Practitioners, whereas Generative Pre-trained Transformer Model Version 4 (GPT-4o) has excelled in image-based tasks, thus suggesting a complementary relationship between the two models. However, their performance in the field of physical therapy, particularly in the Japanese National Examination, remains poorly understood. This study aimed to assess the performance of OpenAI-o1 and GPT-4o in the 59th Japanese National Examination for Physical Therapists (JNEPT) in 2024

Methods

A total of 168 text-only questions were administered to OpenAI-o1, and 23 image-based questions were given to GPT-4o, in a zero-shot prompting format. Accuracy was evaluated by comparing the model outputs with the official correct answers released by the Ministry of Health, Labor, and Welfare. Two faculty members specializing in the National Examination for Physical Therapists reviewed all generated explanations for accuracy.

Results

OpenAI-o1 achieved a correctness rate of 97.0% (163/168 questions) and an explanation accuracy of 86.4% (146/168). In contrast, the GPT-4o attained a correctness rate of 56.5% (13/23 questions) and an explanation accuracy of 52.2% (12/23). OpenAI-o1’s primary explanatory errors involved outdated or incorrect knowledge (13 questions), overly simplified discussions (six questions), and misinterpretation of question intent (three questions). GPT-4o’s most common error type was the misinterpretation of a question’s intent due to difficulties in image analysis (eight questions), along with three instances of knowledge-level inaccuracies.

Conclusions

OpenAI-o1 exhibited high accuracy and solid explanatory quality, indicating strong adaptability to both general and specialized content in physical therapy, and showed potential utility in medical education and remote healthcare support. GPT-4o, while showing enhanced multimodal capabilities compared with previous models, requires further optimization in image-based reasoning and domain-specific training. These findings underscore the promising role of LLMs in healthcare and medical education while highlighting the importance of ongoing refinement to meet the rigorous demands of clinical and educational environments.

## Introduction

Chat Generative Pre-trained Transformer (ChatGPT) has amassed a considerable user base since its initial public release, with applications spanning various fields such as education, business, and healthcare [1]. In the medical domain, the adoption of large language models (LLMs) for healthcare and medical support has gained momentum, with increasing evidence suggesting their potential as decision-support tools in medical education and clinical practice [2-4]. However, implementing LLMs in healthcare requires a cautious approach that takes into account both reliability and ethical considerations [5,6]. Considering these developments, research focusing on the evaluation of the medical knowledge and reasoning capabilities of LLMs, including ChatGPT, is on the rise.

Recent reports indicate that Generative Pre-trained Transformer Model Version 4 (GPT-4o) can pass the examinations for Japanese Medical and Healthcare Professional National Licenses [7]. Furthermore, ChatGPT-4 reportedly achieved an accuracy rate of 80.5% in the Japanese National Examination for Physical Therapists (JNEPT) [8]. Nevertheless, the performances of more recently developed models have not been thoroughly assessed. The latest model, Open Artificial Intelligence Model Version 1 (OpenAI-o1), has garnered significant attention for its remarkable accuracy rate of over 98% in the Japanese National Examination for Medical Practitioners [9], revealing its high-level reasoning capabilities. Although OpenAI-o1 exhibits outstanding reasoning abilities, GPT-4o excels in image analysis and responsiveness. It has thus been suggested that these two models can be used in a complementary manner [10].

Ensuring robust performance is essential for the application of LLMs in the healthcare sector. In this context, the present study evaluated the performance of OpenAI-o1, a new model, and GPT-4o, a multimodal LLM, in a national examination for physical therapists in Japan. Since OpenAI-o1 and GPT-4.0 were trained on data only up to December 2023, this study utilized questions from the 59th JNEPT conducted in 2024, which involved unseen data for these models. In addition to examining the accuracy of the responses, this study evaluated their performance in more detail by eliciting explanatory outputs alongside the answers.

## Materials and methods

Structure and content of JNEPT

JNEPT consists of multiple-choice questions: 160 general questions and 40 practical questions. The general questions cover topics such as anatomy, physiology, kinesiology, pathology, clinical psychology, rehabilitation medicine, clinical medicine, and physical therapy. Practical questions specifically center on physical therapy and require advanced expertise. For instance, some questions include a concise case description and require candidates to identify the most appropriate physical therapy intervention. Both general and practical questions may incorporate visual aids, such as images or tables. The examination included two formats: A-type questions, in which candidates choose one correct option from five, and X2-type questions, in which two correct answers must be selected from five options. While the passing criteria may vary between sessions, an overall score of approximately 60% or higher alongside a minimum percentage of correct responses to practical questions is often required.

Data collection and evaluation

**Figure 1:**
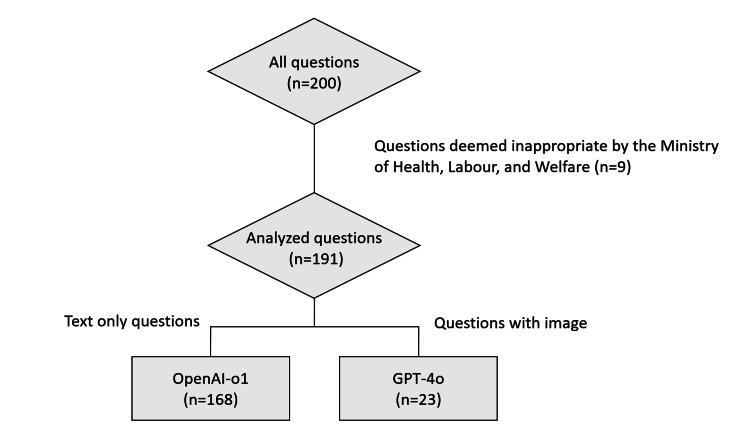
Selection and categorization of questions for analysis GPT-4o: Generative Pre-trained Transformer Model Version 4; OpenAI-o1: Open Artificial Intelligence Model Version 1

This study used questions from the 59th JNEPT [11]. Nine questions that were excluded by the Ministry of Health, Labour, and Welfare owing to inappropriate content were not included in the analysis. A total of 168 text-only questions were input into OpenAI-o1, and 23 image questions were input into GPT-4o using zero-shot prompting to generate answers and explanations (Figure 1).

The zero-shot prompt used for both models was as follows: "I will now input a question from the Japanese National Examination for Physical Therapists. Please provide the correct answer and a detailed explanation." No further customization or fine-tuning of model parameters was conducted. The correctness of the answers generated by the GPT was evaluated against the official correct answers published by the Ministry of Health, Labour, and Welfare [11]. Additionally, the explanations generated by the GPT were reviewed for accuracy by two faculty members specializing in JNEPT. Data were collected between December 10 and 12, 2024.

## Results

Overall, OpenAI-o1 demonstrated a correctness rate of 97.0% (163/168 questions), while GPT-4o's correctness rate was 56.5% (13/23 questions). Specifically, OpenAI-o1 achieved a correctness rate of 84.2% (16/19 questions) for text-based practical questions and 98.7% (147/149 questions) for general questions. In contrast, GPT-4o attained a correctness rate of 57.1% (12/21 questions) for image-inclusive practical questions and 50.0% (1/2 questions) for general questions (Table 1).

**Table 1:** Accuracy rates of OpenAI-o1 and GPT-4o Text-only questions were input into OpenAI-o1, whereas image questions were input into GPT-4o GPT-4o: Generative Pre-trained Transformer Model Version 4; OpenAI-o1: Open Artificial Intelligence Model Version 1

Questions	OpenAI-o1	GPT-4o
Practical questions (n=40)	84.2% (16/19)	57.1% (12/21)
General questions (n=151)	98.7% (147/149)	50.0% (1/2)
Total	97.0% (163/168)	56.5% (13/23)

An analysis of the explanations accompanying the incorrect answers revealed distinct error patterns for each model. Three of OpenAI-o1's five incorrect responses were attributed to "misinterpretation of question intent," reflecting a failure to fully comprehend the provided information. For example, in one case, the model failed to consider the specified rest period and test results detailed in the question, generating answers and explanations solely based on the disease name. The remaining two errors were due to "knowledge-level errors," stemming from inaccurate medical knowledge, such as incorrect understanding of disease mechanisms or outdated kinematic knowledge. The 10 incorrect responses by GPT-4o predominantly involved "misinterpretation of question intent," with seven errors caused by difficulties in interpreting the attached images. These included misinterpretation of anatomical landmarks in medical images and errors in analyzing ECG readings. The remaining three errors were categorized as "knowledge-level errors," similar to those observed in OpenAI-o1, such as inaccurate descriptions of disease characteristics (Table 2).

**Table 2:** Error categorization of incorrect answers and explanations for OpenAI-o1 and GPT-4o The table summarizes error types observed in incorrect answers and explanations for OpenAI-o1 and GPT-4o. Frequencies are shown in parentheses GPT-4o: Generative Pre-trained Transformer Model Version 4; OpenAI-o1: Open Artificial Intelligence Model Version 1

Model	Error in incorrect answers	Error in explanations
OpenAI-o1	Misinterpretation of question intent (3)	Knowledge-level errors (13)
	Knowledge-level errors (2)	Deficiencies in explanation quality (6)
		Misinterpretation of question intent (3)
GPT-4o	Misinterpretation of question intent (7)	Misinterpretation of question intent (8)
	Knowledge-level errors (3)	Knowledge-level errors (3)

The explanation accuracy for OpenAI-o1 was 86.4% (146/168 questions), whereas it was 52.2% (12/23 questions) for GPT-4o. The errors in OpenAI-o1's explanations were grouped into three main categories. The most prevalent were knowledge-level errors, involving the use of incorrect technical terms or reliance on outdated concepts, seen in 13 questions. The second category consisted of deficiencies in explanation quality, characterized by overly simplistic or low-quality descriptions, identified in six questions. The final category, misinterpretation of question intent, involved a failure to accurately grasp the question's intent, leading to errors in three cases (Table 2).

For GPT-4o, the most frequent explanation errors stemmed from misinterpretations of the question’s intent, primarily caused by challenges in interpreting the attached images, accounting for eight errors. Additionally, three knowledge-level errors were identified, consistent with those observed in OpenAI-o1 (Table 2).

## Discussion

This study evaluated the accuracy and explanatory reliability of two AI models: OpenAI-o1 and GPT-4o. A total of 168 text-based questions were input into OpenAI-o1, whereas 23 image-based questions were tested in GPT-4o. OpenAI-o1 showed a high accuracy rate of 97.0%, highlighting its superior performance in text-based reasoning. Conversely, GPT-4o achieved a less satisfactory accuracy rate of 56.5%, despite its enhanced multimodal capabilities. An analysis of the explanations accompanying the incorrect answers revealed distinct error patterns for each model. Among OpenAI-o1's five incorrect responses, three were attributed to "misinterpretation of question intent," while the remaining two were due to "knowledge-level errors." For GPT-4o, the 10 incorrect responses predominantly involved "misinterpretation of question intent," with seven errors caused by challenges in interpreting the attached images and three errors attributed to "knowledge-level errors."

Regarding the quality of explanations, OpenAI-o1 exhibited 13 errors at the knowledge level, six instances of decreased explanatory quality, and three cases of misinterpretation of the intent of the question. In contrast, GPT-4o showed significant misinterpretations of question intent due to errors in image analysis as well as three instances of knowledge-level inaccuracies.

OpenAI-o1 demonstrated exceptional reasoning capabilities, suggesting its potential for effective performance in the medical domain [4,9,10]. Its outstanding performance in the current JNEPT further implies its versatility in a wide range of healthcare-related examinations. Practical questions are tailored specifically to physical therapy and require precise knowledge and advanced reasoning skills [8]. The results indicate that OpenAI-o1 successfully meets these rigorous demands. In addition to its high accuracy rate, the generally favorable quality of explanations further highlights OpenAI-o1's potential for applications in medical education. For instance, it can serve as a tool for providing simulated answers and explanations for national examination questions in the context of student training. However, the identified knowledge-level errors (13 cases) and instances of diminished explanatory quality (six cases) suggest gaps in the model's alignment with the latest medical knowledge and educational perspectives. This limitation is likely due to the nature of LLMs that operate within the scope of their training data [12]. These findings underscore the need to integrate mechanisms to update LLMs with the latest guidelines and evidence for use in medical settings [13].

Furthermore, the high performance of OpenAI-o1 can contribute to addressing knowledge disparities in resource-limited regions [14]. Utilizing telemedicine as a support tool for remote education can enable the provision of medical support and education based on a consistent level of knowledge, even in areas that lack specialists. This would not only reduce the burden on existing healthcare professionals and improve service quality but also help mitigate regional disparities in healthcare access and delivery.

GPT-4o is an enhanced version of GPT-4 with improved multimodal capabilities [15]. Sawamura et al. [8] reported that GPT-4.0 achieved an accuracy rate of only 35.4% for questions involving medical images, suggesting that GPT-4o made progress in handling such content. However, its accuracy rate of 56.5% for image-based questions remains insufficient for practical applications. Kato et al. [16] reported a higher accuracy rate for image-based questions in an examination by a Japanese board-certified physiatrist. This discrepancy may imply a relative scarcity of training data in the field of physical therapy compared with the medical domain. In this study, most of GPT-4o's errors in explanation stemmed from its inability to correctly interpret the intent of questions or images. This suggests that the ability to accurately analyze anatomical findings or kinematic features within images has not yet been fully developed or trained. Similar to OpenAI-o1, GPT-4o may benefit from additional specialized training that focuses on medical image recognition or fine-tuned models tailored to specific domains [13]. This limitation could become a bottleneck for the application of the model, particularly in clinical settings where swift and accurate decision-making is crucial.

Limitations

This study has some limitations that should be noted when interpreting our findings. Firstly, we focused solely on the 59th JNEPT, which may limit the reproducibility and generalizability of our findings in other scenarios. Further evaluation using questions from different years or other certification examinations, such as those for medical practitioners or nurses, is necessary to assess the performance of the model on a broader scale and to evaluate its generalizability across various healthcare contexts. Second, the influence of temperature parameters and prompt design must be considered. The outputs of LLMs highly depend on temperature parameter settings and prompt structures [17,18]. Although this study employed zero-shot prompting, variations in prompt design could potentially affect both the accuracy and quality of the explanations. Therefore, further investigation into the optimal prompt design is required. Third, there is an inherent risk associated with the lack of accountability in LLMs. These models rely on inferences based on their training data and cannot take responsibility for the correctness of their outputs or actions based on them. As shown in this study, errors in answers and explanations cannot be fully eliminated. Consequently, there are clinical and ethical risks associated with relying solely on model outputs for decision-making [6,13].

Addressing these concerns will require a thorough and ongoing discussion that encompasses the entire field. By expanding the scope of research to include additional certification exams and optimizing model parameters, future studies can provide a more comprehensive understanding of these AI models’ capabilities and limitations, ultimately enhancing their applicability in diverse healthcare and educational contexts.

## Conclusions

OpenAI-o1 and GPT-4o demonstrated a certain level of utility in addressing questions in JNEPT. The findings of this study highlight the potential applications of LLMs in the medical and educational fields and simultaneously identify critical challenges for future performance improvements and societal implementation. It is anticipated that feedback from research and practical applications will contribute to the advancement of these models, thereby establishing systems that enable their safe and effective utilization.
